# EHMTI-0094. Effect of nifedipine on memory impairment induced by repetitive spreading depression

**DOI:** 10.1186/1129-2377-15-S1-F16

**Published:** 2014-09-18

**Authors:** M Lotfinia

**Affiliations:** 1 Neuroscience, Shefa Neuroscience Research Center, Tehran, Iran

## Introduction

Spreading depression (SD) is known by transient loss of spontaneous and evoked neuronal activity and changes in ionic, metabolic and hemodynamic characteristics of the brain. It has been shown that repetitive SD produced memory deficits in juvenile rats. Furthermore, the role of Ca2+channels on induction and propagation of SD was investigated by several scientists. The aim of the present study was to study the role of a Ca2+channel-blocker, nifedipine, on memory deficits induced by repetitive SD.

## Materials and methods

Wistar rats (60-80gr) were divided into 4 groups and nifedipine (1 mg/kg) was administrated weekly for 4 weeks in SD group. SD was also induced weekly for four weeks by KCl (2 M). Retrieval of spatial memory was evaluated by T-maze memory test.

## Results

The T-maze test demonstrated that memory was impaired in SD group. The memory retrieval significantly improved by application of nifedipine.

## Conclusions

This study suggests the possible role of calcium channels in memory impairments following repetitive SD.

